# Addressing Value Tensions in the Design of Technologies to Support Older Persons (AgeTech) Using Responsible Research and Innovation and Value Sensitive Design

**DOI:** 10.1007/s11948-025-00541-4

**Published:** 2025-07-01

**Authors:** Nadine Andrea Felber, Wendy Lipworth, Yi Jiao Angelina Tian, Vanessa Duong, Tenzin Wangmo

**Affiliations:** 1 Institute of Biomedical Ethics, Faculty of Medicine, University of Basel, Bernoullistrasse 28, 4056 Basel, Switzerland; 2 Department of Philosophy, Macquarie University, 25B Wally’s Walk, Sydney, NSW 2109 Australia

**Keywords:** Responsible innovation, Value sensitive design, Aged care, Smart homes, Values

## Abstract

The ageing of the global population has prompted the development of many technologies to support older persons (AgeTech). Those developing AgeTech need to not only consider different end users, including older persons and their caregivers, but also be cognizant of the fact that these groups have a variety of, often conflicting, values. The frameworks of Responsible Research and Innovation (RRI) and Value-Sensitive Design (VSD) both emphasize the integration of end users’ values into the process of designing new technologies. Drawing from recent empirical and theoretical AgeTech literature, this article presents an argument for applying these frameworks to the field of AgeTech to successfully identify values and manage tensions among them. It aims to inform a more successful AgeTech innovation process in which new technologies represent and prioritize what their intended end users value.

## Introduction

Populations are growing older globally. By 2050, people aged 65 or older will make up 16% of the world’s population (United Nations Department of Economic and Social Affairs, 2022). International organizations have therefore taken great interest in the promotion of health and well-being of older adults. For example, the Organization for Economic Cooperation and Development (OECD) started promoting the concept of “aging in place” in 1994, while the World Health Organization (WHO) committed to the term of “active aging” in 2002, with its three pillars of health, participation and security (Aceros et al., 2015). Alongside these broad policy initiatives, there has been an intensive focus on developing new caregiving technologies for older persons (also known as AgeTech) in the hope that these may help to “diffuse an age time bomb” (Mort et al., 2015, p. 439).

AgeTech is comprised of a wide range of technologies, the most common of which are wearables, sensors and assistive robots. Wearables alert caregivers when a person experiences a fall (Bet et al., 2019; Chaudhuri et al., 2014), monitor health related data and send it to caregivers (Olmedo-Aguirre et al., 2022) or locate persons with dementia (Chung et al., 2021). Ambient assistive living technologies work through sensors installed in the home, and monitor various health markers, detect abnormalities and alert caregivers when needed (Braspenning et al., 2022; Martin et al., 2023). Such sensors can be supplemented with cameras for further monitoring (Emilsson et al., 2023; Tian et al., 2023). Assistive robots support older persons with tasks of daily living (Bedaf et al., 2019; Fattal et al., 2022), provide social interaction and combat loneliness (Abdi et al., 2018; Bemelmans et al., 2012; Kachouie et al., 2017) or are used in dementia care (Berridge et al., 2021; Hung et al., 2019; Wangmo et al., 2024).

Importantly, there are two distinct (although intersecting) goals of AgeTech: to address the social and economic challenges posed by ageing, and to reduce the associated burden on older persons, and formal and informal caregivers (King et al., 2021). Given that AgeTech does not exist solely for the good of individual end-users, it is important to ensure that AgeTech solutions are implemented for the benefit of end users and “not just for reasons of efficiency and cost-effectiveness …” (Rubeis & Sixsmith, 2024, p. 1). This is challenging for two reasons. First, a focus on cost-effectiveness and efficiency may stand in competition, or even conflict with the notion of “benefit” for end users. Second, there can be multiple end-users of AgeTech, including older persons themselves, formal caregivers and informal caregivers, who may have different visions of what constitutes a “benefit” for them. Therefore, both older persons and caregivers need to be considered when designing and implementing AgeTech (Forsberg et al., 2021; Papadopoulos et al., 2018).

Complicating matters further, there are many different ways of interpreting the notion of “benefit” in this context. For example, it can be understood in abstract ethical terms, in economic terms, in functional terms or in experiential terms. While all these elements of benefit are important in guiding the design and implementation of AgeTech, it is crucial to consider what end-users actually *value*—i.e. “what is important to people in their lives” (Friedman & Hendry, 2019, p. 24). There are several reasons for this. First, values represent fundamental principles and priorities that shape decisions, actions and perceptions of what kinds of improvements are meaningful to people. Unless values are identified and accounted for in the design and implementation of AgeTech, these technologies will not be embraced even if they offer an ostensible “benefit”. Indeed, many technologies have failed to be widely and commonly used in caregiving, despite some of them existing for many years (Felber et al., 2024; Tian et al., 2024). One of the reasons for this gap between existing technologies and lack of use could be that end users do not perceive that they add value to their lives (Ahlin et al., 2023; Pols & Moser, 2009). As Neven observes, when AgeTech fails, this is in part because designers fail to understand what end users perceive as valuable about themselves or in their lives, and therefore do not design technologies that enhance these values (Neven, 2010)

Another reason for articulating values and using them as the basis of AgeTech design is that while values are not fixed, they tend to be relatively stable. Unlike preferences and needs, values are “lasting convictions or matters that people feel should be strived for in general and not just for themselves to be able to lead a good life or realize a good society.” (van de Poel, 2011, p. 72). This is particularly important for AgeTech because older persons’ physical and cognitive abilities, living arrangements, and care requirements are not stable, but the temporal stability of values facilitates long-term planning and evaluation of AgeTech solutions, as well as the creation of adaptable solutions that can change over time, while also remaining aligned with stakeholders’ fundamental principles.

There is now an extensive body of empirical research exploring stakeholders’ values in relation to the design, implementation and use of AgeTech. For example Loveys et al. (2022) investigate in their qualitative study the value of connectedness in virtual conversational agents, a value that only emerged when consulting potential end users. Similarly, in a study of views on assistive robots Papadopoulos et al. (2018) found that caregivers’ focused more on the benefits this technology could have on their care recipients than on themselves, often worrying about the privacy of their care recipients specifically. Such research provides support for our assertion that values and value tensions are key considerations in AgeTech design. In the following, we synthesize our works on values in AgeTech. We then outline two practical frameworks for integrating value considerations into technology innovation—Responsible Research and Innovation (RRI) and Value Sensitive Design (VSD) —and show how they can be usefully applied in the AgeTech context. Finally, we show how the concept of voluntarist reasons (Chang, 2009, 2020; Kozlovski, 2022) can be used to address value tensions in the design and implementation of AgeTech.

## Values and Value Tensions in AgeTech

Between June 2020 and November 2024, we conducted a mixed-methods research project funded by the Swiss National Science Foundation (SNSF) entitled “SmaRt homES, Older adUlts, and caRegivers: Facilitating social aCceptance and negotiating rEsponsibilities [RESOURCE]”. The project consisted of two systematic reviews (Felber et al., 2023; Tian et al., 2024) and a qualitative interview study (60 interviews) (Felber et al., 2024, 2025; Martani et al., 2024; Tian et al., 2024; Wangmo et al., 2024) in which older persons (aged 65 or older), professional caregivers (working in nursing homes or providing home care) as well as informal caregivers (providing care to relatives with or without dementia), were asked about their opinions on wearables, ambient sensors, cameras, socially assistive robots and virtual reality, and if and how they would use these technologies to support their own aging or that of their care recipient. One of the two systematic reviews (Felber et al., 2023) revealed that that the current literature on AgeTech emphasizes a range of values, including privacy, humanness, autonomy, responsibility, the prevention of ageism and stigma, and trust in AgeTech. The centrality of values, and conflicts among them, was confirmed in the qualitative study (Felber et al., 2024, 2025; Martani et al., 2024; Tian et al., 2024; Wangmo et al., 2024).

There were five key findings in the study that are of relevance to values (described in more detail in five published articles). First, when participants’ responses were mapped on to the values of UTAUT (Unified Theory of Acceptance and Use of Technology) such as usefulness (called performance expectancy in the theory) and ease of use (called effort expectancy), values differed among stakeholders. In the case of robots, for example, both caregivers and older persons perceived little value in the current capabilities of robots, preferring human contact instead. However, while older persons paid little attention to ease of use when discussing robots, caregivers had the strong impression that robots would increase their caregiving burden, as they imagined that older adults needed supervision when using them. Thus, ease of use seemed to matter much more to carers than it did to older adults (Felber et al., 2024). Furthermore, some older adults considered robots incapable of paying attention to detail and demonstrate emphatic caregiving, while others thought robots to be potentially better than rude or inattentive caregivers. In both cases, attentiveness was an important value, but there were differences in stakeholders’ perceptions of whether this value would be met through the use of robots (Table 1) (Felber et al., 2024).

**Table 1:** Value tensions in the RESOURCE project

Type	Quote	Publication
Value tension within end user group between human care and friendly care	Older_Person__Nursing_home_10: “The nurse sees when I have a seed, let’s say “sand” [in my eye], she can remove it, but a robot doesn’t see that. That’s what I find, the little things and they are not taken into account. That’s also the important thing. How can it wash my intimate parts? … But it’s just these little things that are also part of it, it’s all human.” Vs. Older_Person_Home_1: “I think that it is actually always better to have a nice Pepper than an unpleasant person. So it depends on the quality of the living individual and unfortunately there are many people in the nursing home who work because of the money and you can see that. And I think that if I had to choose, I would rather have the Pepper than someone who says that the money is good, I’ll do it, but after that, I don’t really give a damn.”	Felber et al. (2024, p. 10, 12)
Value tension within end user group between entertainment and realness	Formal_Caregiver_5: “Virtual reality, I think that’s something…. Of the trips I’ve taken, to have that played out again for me, that would be awesome. Yes.” Interviewer: “So reliving your journeys? “ Formal_Caregiver_5: “Exactly, and then also the positive feelings associated with that, I think that would be really … yes, an entertainment.” Vs. Informal_Caregiver_5: “You can’t breathe in the scent of the flowers or sit in a café and try a local specialty (with VR) … So it’s a travel experience light. And that’s probably also very individual, whether that’s enough for someone or whether it then rather feels like…. ah, I’ve missed my chance now to actually do it, and then it also makes you a bit … yes, sad.”	Felber et al. (2025, p. 5, 8)
Value tension between two end user groups between efficiency and human contact	Professional_Caregiver_7: “Why do I have to measure blood sugar every day? This time is then lost for other things, I can use it more sensibly.[…]I spend so much time to measure fever, to measure blood pressure, to measure weight … Does that really have to be the task of nursing in the future?” Vs. Older_Person_Home_6: “[Caregivers] might be happy if they didn’t have to do that anymore, because a machine does that. But those are the few contacts you still have with a human being. And if that is also taken away by such an instrument, then you are completely isolated.”	Martani et al. (2024, p. 9)
Value tension within an individual between reassurance and privacy	Informal_Caregiver_5: “… if my grandma … falls somewhere in the forest or elsewhere, it would certainly be a great relief if her exact location can be tracked. But on the other hand, it is again very surveilling.”	Tian, Duong et al. (2024, p. 5)
Value tension between two end user groups related to dignity and beneficence	Older_Person_Nursing_Home_9: “It [Paro] is not a being, [its] such a strange thing, and that I can turn it on when I feel like and turn it off again when I have had enough. No, I can’t imagine that [using Paro]. If it is something to give to a child and [the child] has a little fun with it, yes, why not? But no, as an adult, no [Paro is not for me]. Not at all.” Vs. Formal_Caregiver_10: “Yes, I saw that show where they introduced it [Paro] to persons with dementia. And I have to say, that’s absolutely, I think that’s absolutely great. Because I think that’s what you really have to focus on, everything that is still nice and yeah … I don’t think you should question,are we taking them back to childhood or…. And if it brings joy, if you see, they have joy … I mean, you also see it with dolls sometimes. I think that’s good, yes”	Wangmo et al. (2024, p. 4, 6)

Second, when virtual reality was explored as a potential caregiving technology, it emerged that caregivers enjoyed the idea of revisiting old memories, while older persons cared more for real experiences. Interestingly, value tensions were found again within the group of caregivers, with disagreement, for example, about whether a virtual experience of a beloved place adds value to a person’s life or takes away from it due to its artificial nature. In other words, there was a conflict between the value of entertainment and stimulation through the technology and the value of realness (Felber et al., under review).

Third, when data were analyzed through the lens of Tronto and Fisher’s (1990) ‘care theory’, which emphasizes values such as attentiveness, competence and responsiveness, ambivalence was evident. For example, wearables and ambient sensors were seen to enable more attentive caregiving through constant monitoring for some, yet also posed a threat to attentiveness due to potential malfunctioning (thereby creating a false sense of attentiveness) (Martani et al., 2024). Hands-on care was valued by older persons who feared that AgeTech would take personal contact away, while some caregivers appreciated the increased efficiency of their time management thanks to technology that took over monitoring tasks (Martani et al., 2024, Table 1).

A similar discrepancy in end users’ values was found when AgeTech variations were discussed in terms of privacy, but this time between different types of devices. The dimension of informational privacy was valued highly and concerns for data protection were mentioned across all technologies. Nevertheless, we found that ambient sensors (including cameras) garnered concerns about one’s fears of surveillance and the protection of one’s physical space. Depending on the place of installation, such as in the bathroom or in the living room, participants also had varying levels of discomfort tied to how *private* they believe the space should be. On the other hand, smart wearables were notably relevant for the protection of psychological privacy, as they generated alerts, which may interfere with one’s decisions or self-identity (Tian et al., 2024). Compared to their caregivers, we found that only older persons mentioned the feeling of discomfort associated with wearing the wearable technology in public settings, thus valuing their independence (by themselves and as perceived by others). This phenomenon is reasonable, as they were the most likely to be in such situations and be affected by any feelings of embarrassment or stigma. During these considerations, there were also evaluations between the concerns for surveillance or data misuse with the perceived benefits to protect the safety of older persons (see Table 1).

Finally, when pet-like robot Paro was discussed, many participants found the robot infantilizing and therefore were against its use despite potential benefits and wished to protect the dignity of older adults. However, some valued the positive effects of using Paro for people with dementia, for example valuing its calming effects or the perceived joy older adults seemed to experience and were therefore accepting of the technology for this group of potential users despite negative associations attached with its use (Wangmo et al., 2024).

Overall, the RESOURCE project revealed that participants’ values often stood in tension between user groups, within these groups, or even within individuals. Table 1 contains quotes from each publication, where a value tension is expressed, to illustrate the significance of values and value tensions.

The centrality of value conflicts in the RESOURCE study is consistent with other AgeTech research. For example, in a study investigating GPS-locating technology for people with dementia Dahl and Holbø (2012) identified value tensions between privacy of the older person and usefulness for the caregiver (Dahl & Holbø, 2012). Jenkins and Draper (2015) discuss the tension between assistive robots as potential companions of older persons, satisfying the value of companionship, versus them only offering monitoring benefits, protecting the value of human care. Additionally, caregiving robots with potential nudging capabilities were found to cause value tensions between autonomy (mostly of the older person) and other values such as beneficence, efficiency or social connectedness (Draper & Sorell, 2017)

## Theoretical Frameworks to Support the Identification and Management of Values and Value Tensions in AgeTech

Formal research studies such as RESOURCE and other studies cited here are an important source of information about values and value tensions in AgeTech. Formal research cannot, however, be the final word on which values should be considered in the design of specific technologies for specific end-users, and how value conflicts should be addressed, because values are inherently contextual and personal and their significance and prioritization will vary considerably across cultural contexts, individual circumstances, and specific care relationships. Therefore, while formal research provides essential guidance and a starting point for conversations, there is a need for effective ongoing dialogue with specific end-users to understand how values manifest and interact in their particular context, and how value conflicts might best be addressed in practice. Frameworks such as responsible research and innovation (RRI) and Value Sensitive Design (VSD) offer useful approaches to detecting and integrating end-users’ values in the process of technology innovation and design (Taebi et al., 2014).

### Responsible Research and Innovation

Responsible Research and Innovation (RRI) can be understood as an umbrella term for a variety of concepts, theories and methodologies that have developed in response to concerns about the design and governance of innovation and technology (K. E. H. Jenkins et al., 2020). While there are many different articulations of RRI, and ongoing debates about then, four conceptual dimensions have been identified as crucial to RRI: Inclusion, anticipation, responsiveness and reflexivity (Burget et al., 2017). Inclusion is understood as the engagement of societal actors in order to understand what is important to them regarding the innovation (Burget et al., 2017). Anticipation refers to describing and analyzing both intended and unintended impacts the innovation will have. Responsiveness entails transparency and accountability in the innovation process, also facilitating changes when necessary (Stilgoe et al., 2013). And reflexivity asks the involved researchers to critically analyze their own values and beliefs (Stilgoe et al., 2013). Broadly speaking, the goal of RRI scholarship and practice is to align innovation with social and ethical values, preferences and needs, going beyond a short term and mostly economic vision (Timmermans, 2017).

While RRI is not aligned with any specific ethical theory or methodology, it is clear that eliciting context-specific stakeholder values is central to the paradigm. For example, proponents of RRI justify its use on the basis that “[a]ctors can include diverse values and worldviews that form pre-conditions to understand what risks and uncertainties are ethically acceptable, challenge (implicit) drivers of researchers and innovators, and align innovations with society’s expectations.” (Wiarda et al., 2022, p. 65). The dimensions of inclusion and reflexivity are especially relevant when it comes to the integration of values of end users in the innovation process, as the former allows the detection of these values and the latter a critical engagement with them, ensuring that one’s own values do not take precedence over those of the end-users.

With regard to stakeholder engagement as a means of eliciting values, RRI is defined as a process by which stakeholders becomes “mutually responsive to each other with a view on the (ethical) acceptability, sustainability and societal desirability of the innovation process (…)” (Schomberg, 2013, p.63). Stilgoe et al. similarly define RRI as “taking care of the future through collective stewardship of science and innovation in the present” (Stilgoe et al., 2013, p. 1570). What these two definitions have in common is their emphasis on the involvement of society. How this involvement is understood, however, varies across different interpretations of RRI (Timmermans & Blok, 2021). For example, the technology ethicist Van den Hoven (2013) proposes that societal actors should be consulted before the innovation process to determine relevant values. In contrast, Von Schomberg considers the involvement of societal actors as necessary for RRI *throughout* the innovation process and encourages constant debate between society and innovators, in order to steer innovation toward societally desired outcomes (Timmermans & Blok, 2021). In both cases, however, value detection and integration are undertaken before a technology is ready to enter the consumer market.

### Value Sensitive Design

Value sensitive design (VSD) is defined as a “theoretically grounded approach to the design of technology that accounts for human values in a principled and comprehensive manner throughout the design process” (Friedman et al., 2013, p. 56). An underlying premise of VSD is that no technology is neutral. Any design choice promotes certain values and demotes others, which is why designers of technologies need to be aware of the effect their design will have on certain values (Brey, 2010; van Wynsberghe, 2013). As with RRI, VSD is not formally aligned with any particular account of ethics, but its methodologies reveal a strong commitment to eliciting and taking into consideration the values of specific stakeholders in specific contexts.

VSD suggests a range of conceptual, empirical and technical investigations to identify relevant stakeholders, their values, possible tensions between those values, and how the design of an innovation may affect them (Friedman et al., 2017). Some accounts of VSD propose to use a pre-determined set of values as a basis for value detection and refine the list for specific contexts (Friedman & Kahn, 2002). Others defend a bottom-up approach, allowing different values to emerge in different contexts (Borning & Muller, 2012; Dantec et al., 2009). While the first approach has the benefit of identifying values that are widely believed to be important, it may overlook context-specific ones, as well as running the risk of focusing solely on abstract ethical concerns, rather than the full range of values actually held by stakeholders. Bottom-up approaches offer context-sensitivity, but require the involvement of all relevant stakeholders and the capturing of all relevant values in each context (Umbrello & van de Poel, 2021).

There are some examples from AgeTech in which VSD has made it possible to detect early design features that match the values of the end users. In a discussion of navigation technology for people with dementia, preferences regarding size, portability and reliability were found by the interviewed group of informal caregivers before the technology was developed (Kowe et al., 2023). In a research project centered around the development of a night time monitoring system for people with dementia, the VSD approach resulted in professional caregivers accepting the new technology quickly when it was introduced, and in the same caregivers protesting when it was removed after the research project concluded (Schikhof et al., 2010). Köhler et al. (2022) used the VSD framework to create a value-map, showcasing the different interactions between values when applying various assistive technologies for people suffering from cognitive decline. They identified 18 core values and how they stand in competition to each other. For example, privacy often conflicted with safety and the value of caring. While the authors did not suggest design solutions to solve these conflicts, they developed use-cases with already existing technologies to ease the tensions (Köhler et al., 2022).

## RRI, VSD and Value Tensions

There are several different ways in which value tensions and conflicts may arise in technology design. First, as found in the RESOURCE project, different stakeholders can privilege different values. Individuals can also be ambivalent about what they value, holding two opposite affective states towards an issue (Marent et al., 2018; Rothman et al., 2017). According to some definition, ambivalence can be experienced by an individual, or by a group (Ashforth et al., 2014), thereby encompassing conflict among groups, among individuals and within individuals. While ambivalence may sound problematic and even potentially paralyzing, researchers have argued that it actually invites reflexivity and engagement, as it forces stakeholders to look at issues from different perspectives (Marent et al., 2018; Rothman et al., 2017). In the context of RRI, Marent et al., therefore suggest that it is important “to embrace ambivalence rather than seek to resolve contradictions and avoid conflicts” (Marent et al., 2018, p. 139), as this will lead to more realistic and desirable outcomes in innovation.

Unfortunately, while RRI scholars acknowledge and even embrace value tensions (Gianni et al., 2014; Schuijff & Dijkstra, 2020), RRI does not provide clear guidance on how to ‘embrace ambivalence’. Ravesteijn et al. (2014) propose dialogue among stakeholders. However, in case of a tension between economic and non-economic values, from previous research we learn that economic values (such as lower costs, for example) are prioritized over other values, thus undercutting the purpose of RRI (Borch & Throne-Holst, 2021; Yan & Ravesteijn, 2019). In theory, the early inclusion of relevant stakeholders, as well as the steps of anticipation and reflection in RRI, can reduce value tensions from becoming problematic (Vandemeulebroucke et al., 2022).

VSD is somewhat more informative when it comes to value tensions. VSD scholars distinguish between value conflicts, value tensions, and design trade-offs (Friedman & Hendry, 2019, p. 137). A design trade-off means that designing for one value diminishes support for the other value. A value conflict “acknowledges potential opposition among values but leaves open whether their resolution must diminish one in order to support the other(s)” (Friedman & Hendry, 2019, p. 137). Lastly, a value tension is a framing that “allows for solutions to balance each value in relation to the others, such that the adjudication of the tension holds each value intact (Friedman & Hendry, 2019, p. 138). Value tensions, in VSD, are therefore the most productive framing of competing values, in the hopes of finding designs that enable the integration of all stakeholder values.

Sometimes however, as Friedman and Hendry acknowledge, tensions between values may not be resolved by engaging directly with the values themselves. This is in part because there is no clear value hierarchy, and in part because competing values are often incommensurable—i.e. there is no single unit with which multiple items (in this case two competing values) can be measured and compared against each other) (Kozlovski, 2022).

VSD scholars propose two solutions that do not require values to be prioritized, balanced, or weighed and measured against each other: One is to commit to a course of action, rather than an underlying value or set of values (Friedman & Hendry, 2019, p. 143). Their argument is that, while sometimes values clash significantly, the course of action to uphold these values may be able to accommodate both values. The example given is one where fundamental Christians and environmentalists both commit to environmental protection. Both want the same outcome, albeit for very different underlying values. In the case of the value tension in AgeTech between valuing efficiency versus hands-on care (Martani et al., 2024), committing to a course of action that accommodates both values could entail the use of AgeTech to automate certain caregiving tasks (such as measuring blood pressure, blood sugar, etc.), but with the commitment of using the freed time for other hands-on activities, such as physical exercise with the care recipient.

The second strategy proposed by VSD is to wait and see, in the hopes that either new ideas arise or that a value shift will take place. In this regard, it is important to note that while values are more stable than preferences, they are not fixed. While it is not always possible to do nothing until values change, van de Poel suggests developing systems that are adaptable, flexible and robust. Adaptability refers to designers or users being able to make changes to the technology that allow the accommodation of changed values (van de Poel, 2021). Returning to our example above of the caregiver valuing efficiency and the care recipient valuing hands-on care, a change of heart from the caregiver about efficiency may result in her wishing to turn some of the automated features off. Flexibility refers to technologies being able to be *used* differently to adapt to changes in values (van de Poel, 2021). The technology itself has not undergone any design modification, but its users have adapted their behavior as to accommodate a changed value. In the case of the granddaughter first feeling uncomfortable about surveilling her grandmother with a wearable, she may start valuing reassurance and connection to the grandmother more, using the wearable to frequently check in on her. Lastly, robustness refers to a technology still performing its functions despite changed circumstances (van de Poel, 2021). For example, an assistive robot should be able to perform its functions without technical problems even as it or its uses are adapted to changing values. In a study conducted by Gasteiger et al. (2022a), they examined robot Bomy, a companion robot that enabled older persons, among other activities, to play games through a touchscreen on the robot. A lagging screen during the experiment prevented Bomy’s older users from fully enjoying the games it offered, compromising their entertainment, which they valued. While it is useful to have strategies to accommodate changing values, van de Poel’s suggestion does not address the circumstances in which values do not change, or in which changes in values that fail to resolve a tension or cause a new tension.

Other scholars have proposed alternative procedures to solve the issue of value tensions. One is to commit to a normative theory, which will guide the prioritization of the values at stake. While VSD generally refrains from such a commitment, some researchers using VSD have injected different ethical theories into the framework, such as care ethics (Frigo et al., 2023; van Wynsberghe, 2013), or Nussbaum’s capability approach (Jacobs, 2020). However, there are two problems with this approach. First, committing to a normative theory can be as arbitrary as choosing one value over another. The second problem with committing to a normative theory is that it may undermine the underlying rationale of VSD, which precisely proclaims itself as a framework to discover, honor and incorporate different values that emerge in a given context where technology is used or introduced. Especially in the case of AgeTech, with the aforementioned issue of older end-users not being understood well enough to design technologies suitable for them, solving an emerging value tension through a normative theory risks once again de-prioritizing older people’s values. As an alternative to committing to a normative theory Wikström et al. (2024) propose a solution that is both based on quantification. In order to find the best technology to support smart city development in Europe, which is their use case, they refer to so-called key-value-indicators (KVIs) as quantifiable measurements that help showcase the impact of any given future technology. The authors propose to quantify, among other suggestions, the effect of potential future events facilitated by the new technology or processes modified through the technology to compare how a new technology will impact a certain value (Wikström et al., 2024, p. 14). This proposal comes close to solving the issue of incommensurable values mentioned above. Suitable derivates of incommensurable values that are quantifiable can potentially be compared. However, KVIs rely on the successful identification of relevant key values. Furthermore, a comparison of impact on key values through KVIs does not give guidance on how to prioritize competing values. To use the case of smart cities as an illustration, KVIs could potentially showcase brilliantly how a new technology impacts noise or accessibility of public transport in an urban environment. But KVIs do not indicate if noise and accessibility are values held by the impacted end user group and, if yes, which one should be given priority.

## Managing Value Tensions with Voluntarist Reasons

It is not surprising that neither RRI nor VSD offer definitive solutions to the problem of value conflicts in technology design. Indeed, the question of how to resolve competing values (and/or competing moral theories) is one of the core challenge of ethics. Ethics scholarship offers a range of options for addressing persistent value conflict including justifying one set of values (or ethical theory) over another (for example, committing to being a feminist ethicist, a deontologist or a virtue ethicist), applying methods such as ‘reflective equilibrium’ that attempt to find a coherent balance between considered moral judgments and ethical theories, or adopting pluralistic approaches that providing guidance on how to navigate among multiple valid moral frameworks as part of social and political decision-making processes. In practical ethics, it is common to apply ‘problem solving’ methods that recognize that there is no perfect solution and that what matters most is that the downsides of any chosen approach (including failure to support important values) are anticipated and managed.

In practical ethics problem solving methods, there is recognition that at some point it is necessary to find a reason for acting even if no option can be favored rationally over another. These kinds of reasons have been described as ‘voluntarist’ reasons, which are reasons produced from one’s own will that decide in favor of an option over the other, when the preferred types of reasons fail to determine which option we should choose. Put another way, voluntarist reasons “are the reasons we create for ourselves by taking a consideration to be a reason when our given reasons have run out” (Chang, 2009, p. 256). Importantly, while these ‘self-created’ reasons do not emerge from a pre-existing normative framework, neither are they completely arbitrary because they require active engagement with the decision-making context and process, our capacity to commit to certain considerations as reasons, and our willingness to stand behind our reasons.

When competing values cannot be accommodated or balanced, voluntarist reasons might include such considerations as practicality of a particular course of action, the reversibility of a decision and its consequences and the degree to which it keeps options open, or the distribution of benefits and burdens among affected parties. In the case of the competing values of dignity and beneficence in AgeTech, ideally the designers would, together with the older adults consulted for its design, commit to a voluntarist reason to design a robot or a technology that may infringe on one or both values, but is still reasoned and supported (Felber et al., 2022). For example, a less cute looking animal design or a more humanoid design could be chosen, the reasoning behind being that, while dignity and beneficence are incommensurable, the designers and end users nevertheless consider a less children’s toy-like appearance to be more appropriate for older adults. In the case of the competing values of efficiency and hands-on care mentioned above (Martani et al., 2024), an example of a voluntarist reason is to commit to using pill-sorting and reminding technology in light of the scarcity of caregivers, so that the latter can efficiently address more urgent care needs than maintaining every bit of hands-on care possible.

However, even if we were to accept voluntarist reasons as the basis of our decisions on which values to prioritize in design of technologies and commit to that decision, the question remains *whose* voluntarist reasons should be the decisive ones, and what happens if different people’s voluntarist reasons differ from one another. The literature around VSD suggests the group that is impacted the most by the technology should be given priority. In AgeTech, both older persons and their caregivers would be impacted by the different technologies in their caregiving arrangement. However, given that older persons are the primary targeted users of AgeTech, it would be reasonable to give precedence to their values. Indeed, older persons are often the sole focus in articles discussing the design of AgeTech, suggesting that an implicit choice of whose values to prioritize has already been made (Rubeis et al., 2022; Tiribelli & Calvaresi, 2024). Nevertheless, the concept of reflexivity, as embraced by RRI, could foster an internal investigation within each stakeholder as well as a discussion between them and the designers to determine where their voluntarist reasons come from, and which one should be given priority. A way to identify voluntarist reasons and to invite stakeholders to reflect on their values and the priority they give among them is through discursive psychology., an approach that studies how a phenomenon, such as a value, is constructed and performed in discourse (Edwards & Potter, 1992). In a qualitative study involving designers, the use of discursive psychology showed that the values deemed important to the interviewed designers were tied to their identity. Furthermore, the designers offered unprompted justifications why these values are important to them (Cooper, 2023, 2024). This approach therefore seems to invite reflexivity and a potential way to identify voluntarist reasons.

## Case Study: Applying RRI, VSD and Voluntarist Reasoning to AgeTech Design

In this section, we present a case study to better illustrate the our argument that RRI and VSD are useful frameworks for AgeTech design, and that voluntarist reasons provide a way of proceeding when value conflicts cannot be resolved more directly.

Let us assume that a company wants to develop a new caregiving robot for nursing homes that, both provides conversation and entertainment to its users and provides therapeutic assistance, for example guiding through a physical or cognitive exercise program. As the current literature suggests, older persons (Gasteiger et al., 2022a; Neven, 2010) and their caregivers (Arthanat et al., 2020; Papadopoulos et al., 2018) have strong views about such robots (Bedaf et al., 2019; Draper & Sorell, 2017; Felber et al., 2024; S. Jenkins & Draper, 2015). Specifically, as uncovered in our empirical research regarding such assistive robots, values that mattered a lot to caregivers were the ease of use of such robots and how much they would alleviate their caregiving burden (Table 2, quote 1).

**Table 2:** Quotes from published works from RESOURCE showing value tensions for case study

Quote 1	Informal_Caregiver_1: “Yes, but someone has to be there as well, you can’t just put it [robot] down and walk away. I can’t imagine that, someone still has to be there to explain it.” (Felber et al., 2024, p.5)
Quote 2	Professional_Caregiver_4: “That’s really difficult to say. I think it also depends on whether I would live alone or whether there is still a partner, where you are maybe two in this apartment, but that is anyway probably decisive in all these points, whether I would be alone or not.” (Felber et al., 2024, p.6)
Quote 3	Older_Person_Home_9: “Yeah so if you ask me personally then I would say I wouldn’t find that bad. I think it’s a bit of a detachment, a bit of entertainment…. And then it forces you to think a little bit. A little bit, because then maybe he doesn’t give an answer to what you ask him, but to what he has in mind. No, no, why not?” (Felber et al., 2024, p.12)
Quote 4	Interviewer: (Shows video of robot) You’re shaking your head already?Older_Person_Home_2: You get so childish when you’re that old.Interviewer: So childish? What did you say, one becomes childish?Older_Person_Home_2: One becomes childish, yes. So when you’re a kid, you might still enjoy stuff like that, but it’s so unrealistic.Interviewer: Exactly, it’s unrealistic.Older_Person_Home_2: Absolutely no, it’s not like I find it entertaining. It’s not entertaining or educational. Now, if it’s something that can inspire you, then I’ll watch that kind of show, but if it’s just to pass the time, or how do you say it, to kind of entertain you or cheer you up, what … Interviewer: No.Older_Person_Home_2: So I prefer to look for something to read. (Felber et al., 2024, p.10)

Moreover, they perceived the social abilities of the robots as valuable once they were living alone (Table 2, quote 2)

For older persons, there existed a divide between those who would value the entertaining and social aspects of robots and those who found them infantilizing (Table 2, quote 3 and 4)

Also present were divide between older persons who valued human care and those that valued a friendly robot over a human caregiver who was unmotivated or inattentive (Table 1).

In summary, values to be considered, just in this small sample of end users regarding a single technology, were: effectiveness (in the sense that it would alleviate caregiving burden), stimulation (to entertain or combat loneliness), dignity (as the robot should not be infantilizing but may also be perceived as more dignifying than a rude or unmotivated caregiver) and human care (as contact with human caregivers was perceived as more valuable than care provided through the robot). As this short (and not exhaustive) summary already shows, several of these values stand in conflict with each other.

Of special interest in this case, is dignity, firstly because it stands in tension with both stimulation on the one hand and human care on the other, and secondly, because it can be interpreted in different ways, as either dignity that stands opposed to infantilizing treatment or dignity opposed to inattentive or bad human care.

To develop a robot that is capable of navigating these tensions, the company decides to draw upon the principles of RRI and VSD. As suggested by both approaches, the first step is the inclusion of all relevant stakeholders (Burget et al., 2017; Friedman et al., 2017; Wiarda et al., 2022). In our example, this would mean engaging with end users and letting them elaborate further on the values identified in research, hopefully gaining insight on what dignity, stimulation and human care mean to them, where there are tensions, and how these tensions might be resolved. It would also be important to allow new values, that have not been identified in research, to emerge. This could be achieved through a range of different methods, including focus groups, interviews and surveys and inclusion of the RRI framework.

As VSD asks for conceptual, technical and empirical investigations of values, this means that designers and researchers engaging in this topic (e.g. bioethicists) should also be consulted at this stage, the former to provide a technical analysis of how the robot will influence the identified values and the latter to provide a conceptual analysis of the values at stake (Schikhof & Mulder, 2008). This corresponds to the step of anticipation in the RRI framework, where designers try to foresee the impact of their technology and mitigate potential threats to relevant values (Stilgoe et al., 2013).

Iteration is a crucial part of VSD and RRI. This entails that a first prototype that has been developed based on the identified values be given to potential end users for testing (Gasteiger et al., 2022b). Their feedback will then be integrated into the next prototype, which again will be tested by the end users, until the product reaches a stage that satisfies the end users’ values, while at the same time meeting the constraints of the company (such as production cost, for example).

Indeed, realistic behavior of virtual conversational agents seem to foster a feeling of closeness for users, while the issue of the so-called ‘uncanny valley’ did not arise (Loveys et al., 2022). In addition, speech characteristics such as cheerfulness or enthusiasm could be toned down in the robot, as such characteristics could be perceived by older adults as infantilizing (S. Jenkins & Draper, 2015).

Despite these efforts, it is likely that some value tensions will persist. For example, while older adults that worry about infantilization may respond positively to the more humanoid, less chipper robot and wish to promote these qualities further, those older adults that felt their dignity infringed upon by unfriendly or inattentive care may consider such a robot as “rude”, therefore not offering valuable care to them, as it is not more friendly or helpful than their usual care. This is where voluntarist reasons can come into play, as it is not possible to weigh dignity as not being subjected to infantilization and dignity as experiencing friendly, empathetic care objectively against each other and decide which to prioritise. For the end users, both expressions of dignity have value, yet it is not possible to determine which of these expressions of dignity should have more value.

As noted earlier, voluntarist reasons for acting might include such considerations as phow practical a course of action is, its reversibility or how it distributes benefits and burdens among affected parties. Therefore, designers might step back from considerations of dignity and instead focus on which robot design choice would be easiest to develop and implement; which choice would be most easily reversed if resistance proves to be too strong to overcome, or new issues emerge; and which design choice would most fairly distribute risks and benefits among different groups of carers and older persons, taking into consideration issues of equity. For example, the software of the robot is most likely easier to modify then the hardware, therefore the designers may want to focus on software-focused solutions that would be easily reversible if they do not uphold the values of the end users as foreseen or if the relevant values or their expressions change. A practical solution that is also versatile could be adding the option to program the robot with different personalities, letting end users choose their most desired option or to reject these optios. However, this most likely increases both the cost and the complexity of the robot, which then invites the reflection of who will have to deal with these consequences. As shown in our research (Felber et al., 2024), caregivers often worry about an increase in their caregiving work when technology is introduced. An important voluntarist reason could therefore also be the commitment to design a robot that can be easily used by older adults, even if they suffer from cognitive decline.

Importantly, as with any type of practical ethical decision-making, it would be crucial for designers to explain their reasoning—including being transparent about why they have chosen to proceed with voluntarist reasoning, and put in place processes to monitor the outcomes of their decision so that it can be revised as necessary.

## Limitations

The theoretical nature of this article means that our arguments about the utility of RRI, VSD and voluntarist reasoning in AgeTech design need to be tested further in empirical research. It was also beyond the scope of this article to present the details of RRI and VSD frameworks—including their limitations (Burget et al., 2017; Friedman et al., 2013; Friedman & Hendry, 2019; K. E. H. Jenkins et al., 2020; Kozlovski, 2022; Ravesteijn et al., 2014; Stilgoe et al., 2013). For example, RRI and VSD approaches may clash with the economic and time constraints that innovation projects often have (Borch & Throne-Holst, 2021) and may be less useful for examining more mature technologies that are mature. In addition, closely related frameworks to VSD and RRI, such as participatory design (Gasteiger et al., 2022b; Ostrowski et al., 2021), which also emphasize the inclusion of stakeholders in design processes, are not discussed here.

## Conclusion

We have argued for an inclusive and cyclical approach to AgeTech that draws from RRI and VSD frameworks, while also recognizing that value conflicts cannot always be resolved and that voluntarist reasoning may at times be needed. This approach is likely to have resonance with other AgeTech researchers and designers, who have recognized that end users’ values are not consistently integrated well in the design process of AgeTech, thus hindering acceptance (Arthanat et al., 2020; Caldeira et al., 2022; Chen, 2020) and successful uptake of AgeTech in caregiving practices (Ahlin et al., 2023; Austin et al., 2020; Cannizzaro et al., 2020; Nyame-Asiamah, 2020). Other scholars have also argued for greater collaboration between end users and designers (Berridge et al., 2021; Gasteiger et al., 2022b; Ong et al., 2024; Ostrowski et al., 2021) The approach we suggest therefore comes at an opportune moment for fostering more effective innovation in AgeTech.
